# Motion correction using hierarchical local affine registration improves image quality and myocardial scar characterisation from T1 maps acquired with MOLLI

**DOI:** 10.1186/1532-429X-15-S1-P73

**Published:** 2013-01-30

**Authors:** Marcus E Rault, Rashed Karim, Zhong Chen, Tobias Schaeffter, Tobias Voigt, Manav Sonal, Eva Sammut, Christian Buerger, Nick Child, Eike Nagel, Aldo Rinaldi, Reza Razavi, Kawal Rhode, Valentina O Puntmann

**Affiliations:** 1 Imaging Science and Biomedical Engineering, King's College London, London, UK; 2 Philips Healthcare, London, UK

## Background

T1 mapping enables true quantitative assessment of myocardial tissue, separating scars and healthy myocardium. The modified Look-Locker inversion recovery (MOLLI) sequence acquires eleven images over the duration of a minimum of fifteen heart beats to obtain the final T1 map, which is susceptible to undesired respiratory motion due to a relatively long breath-hold. We aimed to implement a motion correction method using a hierarchical local affine registration of MOLLI images and tested if it improved image quality and thus produced more accurate post contrast myocardial tissue T1 estimation.

## Methods

Motion correction was implemented on T1 mapping images acquired 20 minutes post gadolinium contrast injection in 15 patients with a previous history of chronic myocardial infarction. Three experiments were carried out to compare the pre- and post-motion correction images. 1) Comparing visual quality score assessment of the T1 maps by two experienced independent observers; 2) comparing histogram distributions of voxel T1 values within the myocardium to allow tissue characterisation between the scarred region and the non-infarct region; and 3) comparing the "error of mismatch" measured by voxel misalignment in LV endocardial and epicardial borders. Wilcoxon signed rank test was performed to test for significant differences between the median errors of mismatch between the pre and post motion correction images.

## Results

**Figure 1:**
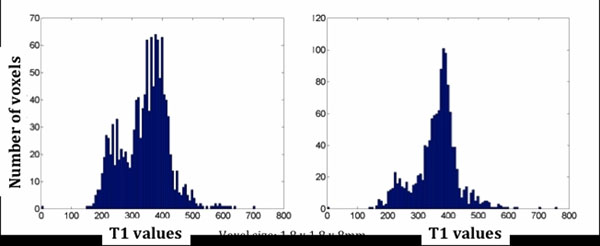
An example of LV myocardial T1 voxel distribution pre motion correction (left panel) vs. post-motion correction (right panel).

**Figure 2:**
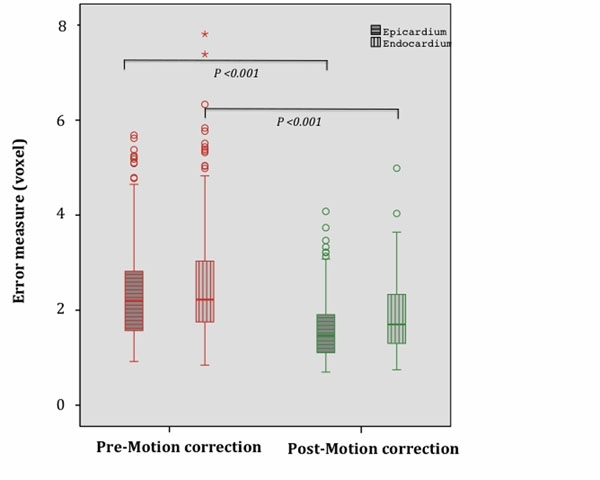
Box plot of measure of error in epi- and endo-cardial borders pre-motion correction (left panel) vs. post-motion correction (right panel).

1) Visual qualitative assessment via two independent observers confirmed motion correction produced an improvement in image quality in 11 out of 15 patients. 2) The histograms assessing tissue characterisation showed an improvement in tissue T1 delineation after motion correction, demonstrated by more distinct signal intensity peaks after motion correction (Figure 1). The estimated post-contrast median T1 values for non-infarct region and scarred region were 375ms (IQR: 60ms) and 240ms (IQR: 10ms); respectively. 3) There was a significant difference in the median measure of errors in epi- and endocardial borders after motion correction (Figure 2). Pre-motion correction: 2.2 voxel (IQR: 1.3) epi; 2.2 voxel (IQR: 1.3) endo. Post-motion correction: 1.5 voxel (IQR: 0.8) epi; 1.7 voxel (IQR: 1.0) endo; p<0.001.

## Conclusions

Motion correction using hierarchical local affine registration yields better imaging quality thus allowing more accurate tissue characterization from T1 maps acquired with MOLLI sequence.

## Funding

n/a

